# Low resting metabolic rate and increased hunger due to β-MSH and β-endorphin deletion in a canine model

**DOI:** 10.1126/sciadv.adj3823

**Published:** 2024-03-06

**Authors:** Marie T. Dittmann, Gabriella Lakatos, Jodie F. Wainwright, Jacek Mokrosinski, Eloise Cross, I. Sadaf Farooqi, Natalie J. Wallis, Lewis G. Halsey, Rory Wilson, Stephen O’Rahilly, Giles S.H. Yeo, Eleanor Raffan

**Affiliations:** ^1^ MRC Metabolic Diseases Unit, Wellcome-MRC Institute of Metabolic Science, University of Cambridge, Cambridge, UK.; ^2^ University of Cambridge Metabolic Research Laboratories and NIHR Cambridge Biomedical Research Centre, Wellcome-MRC Institute of Metabolic Science, Addenbrooke’s Hospital, Cambridge, UK.; ^3^ Department of Physiology, Development and Neuroscience, University of Cambridge, Cambridge, UK.; ^4^ School of Life and Health Sciences, University of Roehampton, London, UK.; ^5^ Department of Biosciences, Swansea University, Swansea, UK.

## Abstract

Mutations that perturb leptin-melanocortin signaling are known to cause hyperphagia and obesity, but energy expenditure has not been well studied outside rodents. We report on a common canine mutation in pro-opiomelanocortin (*POMC*), which prevents production of β–melanocyte-stimulating hormone (β-MSH) and β-endorphin but not α-MSH; humans, similar to dogs, produce α-MSH and β-MSH from the *POMC* propeptide, but rodents produce only α-MSH. We show that energy expenditure is markedly lower in affected dogs, which also have increased motivational salience in response to a food cue, indicating increased wanting or hunger. There was no difference in satiety at a modified ad libitum meal or in their hedonic response to food, nor disruption of adrenocorticotropic hormone (ACTH) or thyroid axes. In vitro, we show that β-MSH signals comparably to α-MSH at melanocortin receptors. These data implicate β-MSH and β-endorphin as important in determining hunger and moderating energy expenditure and suggest that this role is independent of the presence of α-MSH.

## Teaser

A common mutation in pet dogs shows that the POMC-derived peptide β-MSH has an independent role in controlling energy homeostasis.

## INTRODUCTION

Body weight is subject to homeostatic control, influenced by environmental and genetic factors. The central leptin-melanocortin axis is a critical nexus for energy homeostasis, integrating signals that indicate systemic energy status and translating them into behaviors and physiological changes that moderate food intake and energy expenditure. Hypothalamic neurons expressing pro-opiomelanocortin (*POMC*) are activated in response to nutritional excess and inactivated by nutritional deficiency (*1*, *2*).

*POMC* is posttranslationally modified to produce a number of neuroactive peptides, which are released when *POMC* neurons are activated. These include the closely homologous peptides α–melanocyte-stimulating hormone (α-MSH) and β-MSH, which are activators of melanocortin receptors (MC3R and MC4R), and the mu agonist β-endorphin (*3*). Activation of MC4R leads to reduced food intake and increases energy expenditure (*2*). MC3R appears to primarily regulate the disposition of energy into growth and reproduction, having a role in timing of sexual maturation and accumulation of lean mass (*4*).

We previously reported a mutation in *POMC* in Labrador and flat-coated retriever (FCR) dogs to be a major modifier of body weight and adiposity (*5*). The 14–base pair (bp) deletion at position 17 (19431807 to 19431821) causes complete disruption of β-MSH or β-endorphin production from affected alleles but does not affect the sequence of α-MSH. Here, we studied affected dogs with the aim of understanding how POMC-derived ligands affect energy homeostasis. The work is timely given that MC4R agonist drugs have recently been approved for the treatment of several disorders including genetic forms of obesity, hypoactive sexual desire, and some skin conditions (*6*–*10*).

Human patients with severe, early onset obesity caused by homozygous or compound heterozygous *POMC* mutations were first reported in 1997 (*11*, *12*). Although those patients bore mutations, which prevented production of α-MSH, β-MSH, and β-endorphin, causality tended to be placed on α-MSH absence. This is because the dominant research comes from studying rodents that lack a critical proteolytic cleavage site in POMC and function normally with only α-MSH, never producing β-MSH (*3*).

However, humans and dogs produce both α- and β-MSH. In humans, loss of functional α-MSH with preserved β-MSH has been reported and data suggest that loss of α-MSH is sufficient to cause obesity, although variants do not consistently segregate with obesity in families or populations (*13*–*16*). Recent evidence that β-MSH and desacetyl α-MSH are produced in considerable excess of acetylated α-MSH in the human hypothalamus supports older data from immunofluorescence experiments (*17*) and suggest that β-MSH is also likely to be physiologically important in humans (*18*).

Patients with *POMC* mutations that result in aberrant forms of β-MSH and reduced MC4R activation have been identified, and the variants also shown to cosegregate with obesity in the probands’ families (*14*, *17*, *19*). The same variants were more common in severely obese patients but were not absent from normal weight controls, meaning the importance of β-MSH remains debated.

The second peptide affected by the retriever *POMC* mutation, β-endorphin, also plays a role in energy homeostasis. β-Endorphin knockout mice are hyperphagic and obese but have normal metabolic rate (*20*). Tests of the hedonic and motivational aspects of feeding suggest that β-endorphin signaling preferentially supports feeding driven by palatability, but not incentive motivation (*21*), and more recent work implicates it as a factor that regulates hunger driven by ghrelin’s action on Agouti-Related Protein neurons (*22*). That is consistent with the general understanding of *POMC* neurons as anorexigenic but complexity is introduced because a subset of *POMC* neurons that express CB1R cause an increase in food intake on stimulus of those receptors in the sated state, an effect that is partly mediated by β-endorphin (*23*). Overall, the majority of the data points toward β-endorphin absence as likely to promote increased hunger and affect the hedonic response to food.

The retriever mutation in *POMC* causes higher owner-reported food motivation, but we had no information on energy expenditure, nor rigorous measures of eating behavior (*5*). The mutation has an allele frequency of 12% in Labradors and 60% in FCR and therefore presents an opportunity to systematically study the physiological and behavioral consequences of deficiency of these peptides in a naturally occurring animal model.

Here, we report the results of extended studies of eating behavior and energy expenditure in pet dogs affected by the retriever *POMC* mutation that further establish the effect of this mutation on energy homeostasis. We show that affected dogs have higher motivational salience in response to a food cue and lower resting energy expenditure and examine implicated neuroendocrine mechanisms. These data implicate β-MSH as playing an important role in determining hunger and moderating energy expenditure, and that this role is independent of the presence of α-MSH.

## RESULTS

### The effect of POMC on adiposity, weight, and eating behavior is replicated in FCR

**Fig. 1.**
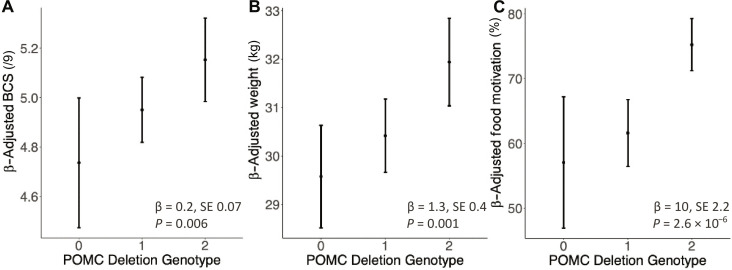
The *POMC* deletion causes increased BCS (obesity), body weight, and owner-reported food motivation in a replication cohort of FCRs. Partial regression plots showing predicted mean and 95% confidence interval (**A**) BCS (*n* = 221), (**B**) weight (*n* = 218), and (**C**) food motivation score (*n* = 180) by genotype after linear regression adjustment for age, sex, and neuter status are shown with mean effect size (β), SE, and *P* values overlain.

In our initial report, we showed that Labradors carrying the *POMC* mutation had greater adiposity, weight, and food motivation but only tested the effect on weight and food motivation in FCR (*5*). New data from an expanded cohort include information on body condition score (BCS), a well-validated measure of canine adiposity based on haptic and visual cues and scored on a nine-point scale on which each point increase represents an ~8% increase in body fat mass (*24*, *25*). These new data confirm that the mutation increases BCS in FCR (*P* = 0.006; mean effect size per deletion allele, 0.2 BCS point). This effect size is smaller than in Labradors, a particularly obesity prone breed, in which we previously reported a 0.5 BCS point increase per deletion allele (*5*, *26*). In newly recruited FCR, we replicated the previously reported association of the mutation with body weight (effect size of 1.3 kg per deletion allele; *P* = 0.001, *n* = 218) and food motivation as reported by owners regarding behavior in the home (effect size of 10%; *P* = 6.3 × 10^−6^, *n* = 180) (Fig. 1) (*27*).

### Dogs heterozygous for the retriever POMC mutation had increased hunger

We hypothesized that β-MSH deficiency would result in behaviors that promote food intake (increased “wanting” behavior and greater ad libitum food intake) and that β-endorphin deficiency would reduce affected dogs’ hedonic response to food (“liking”). We recruited pet, adult, healthy Labrador dogs, which were either healthy weight or modestly overweight but not obese (BCS of 4 to 7/9). Dogs were either heterozygous or wild type for the mutation. Their eating behavior was tested under standardized conditions.

**Fig. 2.**
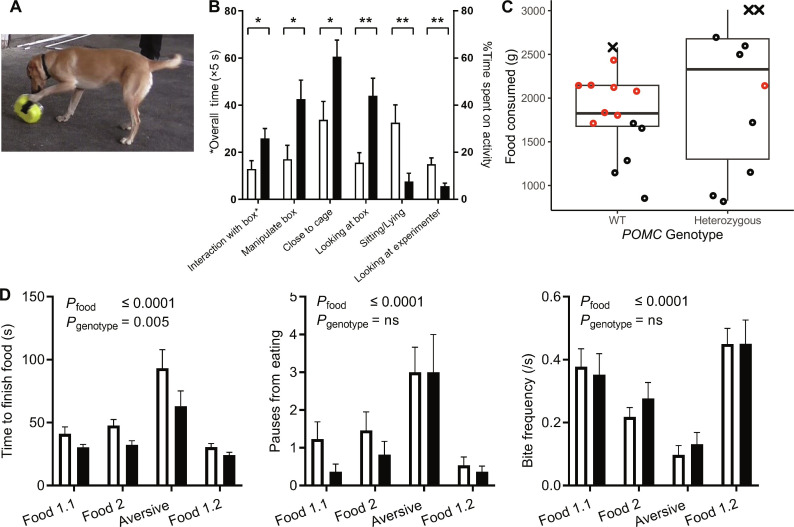
The *POMC* mutation increases wanting/hunger for food, but not satiety or “liking.” (**A** and **B**) Incentive salience in response to a food cue was measured using an inaccessible food test. Behaviors directed at the inaccessible food (e.g., biting, pawing, or otherwise manipulating the box, staying close to it, or looking at it) occurred for longer in dogs heterozygous for the mutation, and they spent less time resting or interacting with the experimenter. This test is a measure of wanting (hunger). *P* values for unpaired *t* tests indicated (*, <0.05 and **, <0.005). Left: *Y* axis refers to overall time spent interacting with box (observer 2, blinded to genotype). Right: *Y* axis relates to percentage time spent on individual behaviors (observer 1). (**C**) Food intake at a modified ad libitum meal was high irrespective of *POMC* genotype, but there was no significant difference between wild-type and heterozygous dogs. Significantly more wild-type dogs vomited or regurgitated (indicated by red circles) at the termination of the study (*t* test, *P* = 0.01). Points shown with “X” identify two dogs in which the trial was stopped at the maximum (2940 g) intake set in the ethical approval and one dog where feeding was stopped at the request of the owner. (**D**) Eating behavior in response to foods of differing palatability. Dogs affected with the mutation retained discrimination between appealing and aversive foods but ate more quickly. Graphs show mean and SEM; white, wild type (WT); black, heterozygous mutation. *P* values for two-way analysis of variance (ANOVA) are shown. ns, not significant.

To test “wanting,” we applied an inaccessible food test to measure incentive salience in response to food. Three hours after a standard meal, a sausage was shown to the dogs and then sealed in a perforated plastic box, after which the dogs’ behavior was recorded. Dogs heterozygous for the mutation showed greater incentive salience in response to a food stimulus, spending significantly more time interacting with the box and less time exploring/resting (Fig. 2A and movie S1), showing greater hunger in dogs with the mutation.

This finding is consistent with reports of extreme hunger in human patients with *POMC* mutations (*28*) and is likely to underlie increased food intake by driving behaviors such as food seeking, stealing, begging, and foraging, consistent with owner-reported data from our previous paper where there food motivation increased additively with additional copies of the deletion (*5*).

### Heterozygosity has no effect on satiety or the hedonic response to food

To test food intake, we used a modified ad libitum meal. After standardized pretest feeding and a 14-hour overnight fast, dogs were offered a standard amount of canned dog food every 20 min until the dog either failed to finish the food offered or vomited/regurgitated. Dogs across both groups ate a mean of 1.9 kg of food (wild type: 1.8 kg, 333 kJ/kg; heterozygous: 2.1 kg; 336 kJ/kg), but there was no significant difference between the genotype groups (Fig. 2B). Vomiting or regurgitation occurred significantly more frequently in wild-type dogs (9 of 16) than heterozygous dogs (1 of 12) at the end of the feeding period (*P* = 0.01).

These data suggest that satiety is not impaired by heterozygous depletion of β-endorphin and β-MSH, although food intake was remarkably high even in wild-type dogs—on average, they ingested twice their total daily energy requirement (mean, 2.0 times; range, 0.8 to 3). Vomiting or regurgitation terminated the trial in more than half of the wild-type dogs but only one heterozygous dog. This may reflect a genetically driven difference in tolerance of large meals, which (because vomited food is not assimilated) would serve to increase the final energy intake in affected dogs.

Testing homozygous dogs may have provided greater power to detect a difference between genotypes if one exists, as might be suggested from data that shows that *POMC* mutations only cause human monogenic obesity in the homozygous state (*29*), but recruiting sufficient homozygous Labrador subjects was not possible (only 4% of Labradors are homozygous). However, even if a true difference exists, dog owners usually restrict meal size to considerably less than even the wild-type dogs consume, meaning that altered satiety is not implicated in obesity associated with the mutation.

To examine whether the mutation altered the hedonic response to food, dogs were offered small amounts of two commercially available dog foods (food 1 and 2) and one of those foods with an aversive flavoring (lime juice). The time to eat the food and behaviors displayed were recorded using video coding. When presented with aversive flavored food, all dogs paused more often, took fewer bites, and took longer to eat. Dogs with the *POMC* mutation consumed the food more quickly than wild-type dogs but discriminated between the foods to the same extent (Fig. 2D). There is no evidence of an altered hedonic response to food because of the mutation. However, we acknowledge that this test, the best available for use in pet dogs, was not a sophisticated measure of hedonic response.

### The Retriever POMC mutation results in lower resting metabolic rate

We hypothesized that dogs with the mutation would have lower resting energy expenditure. To test this, pet FCR dogs, wild type or homozygous for the mutation, aged 2 to 7 years old, in good health and of lean body condition were selected. FCR dogs were selected because we hypothesized that the effect size would be subtle, and the mutation is more common in this breed. After a period of standardized feeding and habituation to the experimental chamber, energy expenditure measurements were taken in the post-absorptive state from dogs at rest using indirect calorimetry using a flow-through system.

**Fig. 3.**
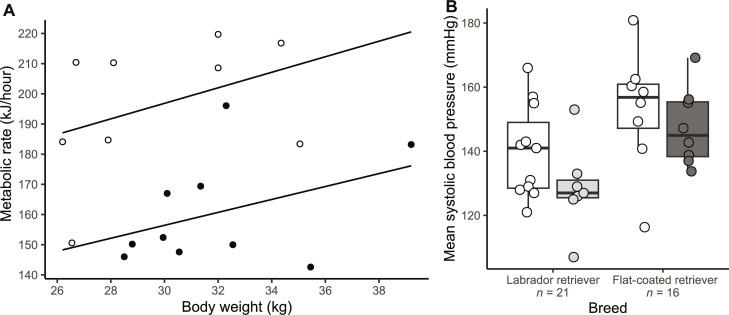
Metabolic rate and systolic blood pressure were lower in dogs with the *POMC* deletion. (**A**) Energy expenditure measured in FCR dogs at rest using indirect calorimetry. ANCOVA showed dogs homozygous for the mutation (black circles) had significantly lower resting energy expenditure compared to wild-type dogs (open circles) while adjusting for body weight. Linear regression lines for each genotype group are shown. (**B**) Mean systolic blood pressure was lower in Labradors heterozygous for the *POMC* mutation. Systolic blood pressure measured in dogs at rest was significantly lower in Labradors with the mutation (wild type: mean, 140 mmHg; heterozygous: mean, 128 mmHg; *P* = 0.003 in regression model including age, sex, BCS, and weight). A similar trend was not significant in FCR (wild type: mean, 153 mmHg; homozygous: mean, 148 mmHg; *P* = 0.5). Normal systolic blood pressure in dogs is 110 to 160 mmHg (*33*). White, wild type; light gray, heterozygous; dark gray, homozygous deletion.

Analysis of covariance (ANCOVA) showed that dogs homozygous for the mutation had significantly lower resting energy expenditure (*F*_1,16_ = 16.85, *P* ≤ 0.001) compared to wild-type dogs while adjusting for body weight. Linear regression modeling was performed, initially including *POMC* genotype, weight, age, sex, BCS, duration resting at time metabolic rate (MR) reported, and average daytime temperature for the month before measurement, reducing stepwise until the following minimal model below was reached: resting metabolic rate (kJ/hour) = (β_wildtype_ × weight in kg) + (β_homozygous*POMC*_ × 1) + *C*, in which *C* (SE, *P* value) = 85.3 (44.1, 0.07), β_homozygous*POMC*_ = −40.7 (9.21, <0.001) and β_wildtype_ = 2.36 (1.37, 0.10), model *R*^2^ = 0.55 (*P* = 0.002) (Fig. 3A). There was no difference in respiratory quotient (RQ) between genotypes (both groups mean RQ of 0.86).

This means resting energy expenditure is approximately 25% lower for adult weight dogs—large enough to significantly decrease how much food is required to maintain healthy body weight. With increased hunger-driven food seeking, this explains the obesity association observed with this mutation. Although large, the effect size is comparable to existing data from other species. Seven human patients heterozygous for mutations causing only α-MSH (*13*) or only β-MSH deficiency (*14*) had normal resting energy expenditure. In contrast, two children with homozygous mutations in *POMC* that predicted to prevent production of both α- and β-MSH had an energy expenditure approximately 20% lower that a predicted value, although the authors commented that their predictions may not be reliable in this extreme childhood obesity (*12*). *POMC*^−/−^ mice show a 22% reduction in energy expenditure (*30*), but mice lacking β-endorphin have normal energy expenditure (*20*).

Together, these data robustly implicate β-MSH deficiency in causing the reduced energy expenditure observed in dogs bearing the *POMC* mutation. Affected dogs are clinically healthy with no evidence of hypometabolism affecting neurological or other organ system function, suggesting that this lower metabolic rate may reflect increased metabolic efficiency that would be an appropriate, if mechanistically unclear, adaptation to the physiological signal of energy deficiency that is implied by deficient melanocortin signaling (*31*).

### The Retriever POMC mutation results in lower blood pressure in Labradors

Because MC4R agonism is integral to the hypertension seen with obesity in other species (*32*), we compared resting systolic blood pressure in dogs with and without the mutation (Fig. 3B). In 21 Labrador retrievers, dogs heterozygous for the mutation had significantly lower systolic blood pressure than wild-type dogs (*P* = 0.003 in regression model including age, sex, BCS, and weight). A similar trend was not statistically significant in a smaller group of 16 FCR (*P* = 0.5).

Situational hypertension may underlie the higher overall results recorded in FCR, which were tested in the clinical research facility, as opposed to Labradors that were tested in their home environments (*33*). Anxiety-induced increase in sympathetic tone, bypassing MC4R mediated pathways, may also explain a blunted effect of carrying the *POMC* mutation in FCR.

Most recordings were within the normal range irrespective of genotype [normal range of systolic blood pressure in dogs is 110 to 160 mmHg (*33*)], expected given that most dogs were of a healthy weight or only modestly overweight (mean BCS Labradors of 5.3/9 and FCR of 5.1/9) (*33*). The lower systolic blood pressure seen in Labradors may indicate a reduction in MC4R-mediated sympathetic tone at play even in healthy weight individuals that may have been previously unrecognized in tests in humans or mice with MC4R mutations where subjects were universally obese (*34*–*36*).

### No evidence that the mutation affects proximal POMC-derived neuropeptides or thyroid hormones

This mutation is a frameshift occurring late in the *POMC* transcript so is predicted not to affect production of α-MSH or adrenocorticotropic hormone (ACTH) (the precursor molecule for α-MSH during *POMC* cleavage) while preventing production of both β-MSH and β-endorphin. However, we considered whether the mutation might cause nonsense-mediated decay of the mutated transcripts, meaning that observed physiological effects might be the result of low α-MSH. Central hypothyroidism has been inconsistently but repeatedly reported in human patients with *POMC* mutations (*15*, *37*) and is present in *POMC* knockout mice (*30*), so we also tested the thyroid axis in dogs of contrasting genotype.

All dogs in the study were healthy and free of clinical signs, consistent with ACTH deficiency or hypothyroidism. Blood samples were drawn from 11 dogs of contrasting genotypes. Serum biochemistry showed normal electrolytes, urea, and other routine analytes, and there were no significant abnormalities on complete blood count. There was no significant difference in thyroxine (T_4_) and thyroid-stimulating hormone (TSH) measured in 11 dogs of contrasting genotypes (table S1). These data show no evidence of cortisol deficiency or hypothyroidism.

We were unable to distinguish α-MSH and β-MSH in canine plasma using antibodies to human or murine peptides or with mass spectrometry, but a validated canine ACTH immunoassay showed no difference between ACTH concentration in plasma samples tested from wild-type, heterozygous, and homozygous deletion dogs (table S1). Because α-MSH is derived from ACTH, this is evidence that dogs with the mutation do not have depletion of α-MSH, only β-MSH and β-endorphin.

### Β-MSH activates metabolic melanocortin receptors to a similar extent to α-MSH

There is only very limited data that quantifies melanocortin receptor activation by β-MSH (in contrast to α-MSH) (*17*). Because altered receptor activation could be a way in which these structurally similar peptides have physiologically independent effects, we sought to investigate this. MCRs are G protein–coupled receptors, which respond to activation with downstream signaling that results in production of the second messenger cyclic adenosine 3′,5′-monophosphate (cAMP) and by recruiting β-arrestin to the plasma membrane. We tested both in vitro by expressing human or canine MC3R and MC4R and measuring the response to stimulation with α-MSH and β-MSH in comparison to a synthetic ligand NDP–α-MSH.

**Fig. 4.**
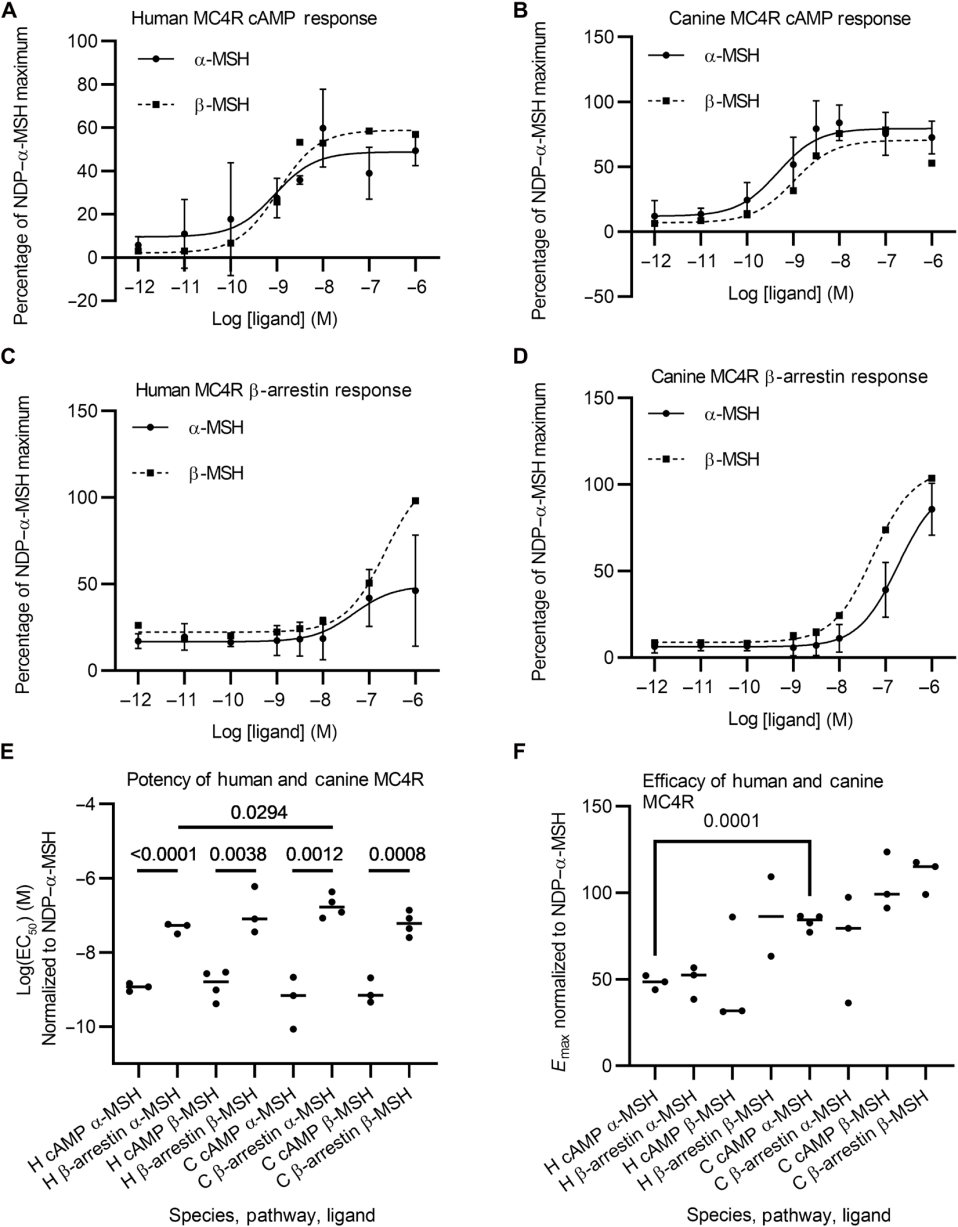
α-MSH and β-MSH activate melanocortin 4 receptor similarly in both dogs and humans. A luciferase based GloSensor cyclic adenosine 3′,5′-monophosphate (cAMP) assay was used to measure MC4R activation in transiently transfected human embryonic kidney (HEK) 293 cells. The dose response following stimulation with α-MSH and β-MSH is expressed in comparison to that in response to NDP–α-MSH for both human (**A**) and canine (**B**) MC4R (both *n* = 3). Coupling between MC4R and β-arrestin was monitored using a NanoBiT protein:protein interaction assay in HEK293 cells transiently transfected with MC4R and luciferase activity following activation by α-MSH and β-MSH compared to that with NDP–α-MSH for both human (C) and canine (D) receptors (both *n* = 3 or 4). (**E**) EC_50_ (median effective concentration) values of each assay to compare potency of α-MSH and β-MSH: individual points plotted with line at median value. Statistical values displayed are unpaired Student’s *t* tests (*n* = 3 or 4). (**F**) *E*_max_ values for each assay. Individual values with median lines plotted (*n* = 3 or 4). Statistical values displayed are *P* values from unpaired Student’s *t* tests.

Consistent with their structural similarity, we showed that α-MSH and β-MSH produced similar activation of these receptors and that canine and human receptors responded similarly to each ligand (Fig. 4 and fig. S1). For β-arrestin recruitment, β-MSH is more potent in both species. These data further support the importance of β-MSH in melanocortin signaling.

## DISCUSSION

Experiments on this spontaneously occurring, pet dog model of obesity confirm the obesogenic effect of a common canine *POMC* mutation, which is carried by Labradors and FCR, and show that its effects are mediated both by increased hunger and lower energy expenditure.

This is a rare example of a common genetic variant affecting obesity risk by not only increasing food intake but also significantly reducing energy expenditure. It is interesting to speculate on the molecular mechanisms underlying this reduced resting energy expenditure. The lack of evidence for a difference in RQ in our data suggests that altered carbohydrate/fat substrate utilization is not responsible. Variation in mitochondrial efficiency is recognized in other species (*38*, *39*) and might be implicated here; this metabolic adaptation might be a sensible physiological adaptation to starvation (*40*, *41*).

The increased motivational salience in response to a food cue by dogs with the *POMC* mutation indicates greater wanting of the incentive (sausage). We suggest that this greater appetitive motivation reflects greater hunger, as reported by human patients with *POMC* mutations (*28*). However, hunger, an interoceptive signal universally recognized by humans as the desire for food coupled with physical discomfort, cannot be directly measured in animals. Rather, persistence in pursuit of food, as measured here, is commonly used as a surrogate measure. This goal-directed behavior is orchestrated in the mesolimbic dopaminergic circuitry (*42*) and is influenced by melanocortin receptor agonists (*43*). Increased wanting likely underlies the well-recognized bias toward engaging in food seeking and eating behaviors in food-deprived animals (*44*).

This greater wanting/hunger is likely to explain the increased food seeking behavior in the home environment that we previously reported in affected dogs (*5*). In the pet home, it may drive greater food intake when owners succumb to begging behavior or dogs are more likely to steal or scavenge food. We hypothesize that if we offered ad libitum food over a protracted period, then affected dogs would return to eat more quickly after a meal and therefore increase their overall food intake, but that experiment was not possible in these pet dogs. Nevertheless, the greater wanting/hunger and owner-reported food motivation is sufficient to predispose dogs with the mutation to obesity.

To maintain a healthy body weight, owners of affected dogs must restrict food intake to below that which would maintain a healthy body weight in wild-type dogs because of their lower energy expenditure. This is challenging in these highly food-motivated dogs. Many slim, affected dogs in this cohort attest to the fact it is possible, if hard work, to achieve this.

We showed that β-MSH activates MC3R and MC4R similarly to α-MSH. This invites us to consider how loss of one of two such similar ligands due to this mutation might generate the physiological effect we report. A “dose effect” may be responsible because only one of the two ligands are produced from affected transcripts. However, multiple lines of evidence point to an as-yet ill-defined complexity in how different *POMC*-derived peptides are stored, trafficked, and released: A study in mouse cells (lacking β-MSH) showed that β-endorphin and α-MSH were stored and released separately (*23*); β-MSH is produced in excess of α-MSH in the human brain (*18*), and clear heterogeneity has been identified in hypothalamic *POMC* neurons (*45*). This nuance may underlie why dogs lacking β-MSH but not α-MSH have the clear, measurable phenotype reported.

The well-studied importance of *POMC*-derived peptides as agonists at melanocortin receptors means β-MSH absence is strongly implicated in driving this phenotype. Normal energy expenditure in β-endorphin knockout mice (*20*) contrasts with multiple reports of reduced energy expenditure when the melanocortin pathway is disrupted (*1*–*3*), and so we suggest that β-MSH absence is responsible for the low-energy expenditure observed in affected dogs. It remains possible that β-endorphin deletion is partly responsible for the hunger phenotype. Lower systolic blood pressure in β-MSH–deficient dogs suggests that MC4R tone may have a role in regulating blood pressure even in the absence of obesity.

We acknowledge that we have not directly measured a-MSH levels, as doing so would require hypothalamic samples from Labradors of the appropriate genotype. However, the dogs we work with are all kept as pets, which precludes us from doing this. However, it is unlikely that α-MSH is depleted in affected dogs for the following reasons. First, while ACTH is not a perfect surrogate for hypothalamic α-MSH, normal concentrations in dogs with the mutation are strong evidence that the mutated transcript does not undergo nonsense-mediated decay following transcription and instead is translated into a POMC protein product, with cleavage sites unaffected, thus enabling production of ACTH at normal circulating concentrations. Production of ACTH in the anterior lobe of the pituitary is dependent on the activity of the prohormone convertase 1/3, and normal concentrations suggest that this cleavage process is intact (*3*). In the pars intermedia of the pituitary, skin, and the hypothalamus, the ACTH undergoes further cleavage and processing active α-MSH (*3*). The authors do not see a compelling reason why, when affected dogs are effectively producing ACTH with normal peptide sequence, these further processing steps to produce α-MSH would be disrupted. Second, affected dogs have no signs of α-MSH deficiency related to its other key receptors, melanocortin receptors 1 and 5 (i.e., they have normal hair and skin pigmentation and normal sebum production) (*11*, *46*).

Combined, canine physiological data, evidence that β-MSH signals effectively through MC4R, and the association of β-MSH deficiency with human obesity (*14*, *17*, *19*) provide evidence that β-MSH acts alongside and independently from α-MSH as a regulator of energy homeostasis in species such as humans and dogs, which produce both α-MSH and β-MSH.

## MATERIALS AND METHODS

### Canine experimental model

This work was approved by the Ethics and Welfare Committee of the Department of Veterinary Medicine, University of Cambridge (CR73, CR125, CR176, and CR276). Dogs studied were companion animals kept as pets in the United Kingdom. The *POMC* mutation is more prevalent in FCR, but this breed is much less common than Labradors, so it is harder to recruit. In addition, FCR are at particular risk of gastric dilation and volvulus, a life-threatening condition that has been associated with rapid eating and large meals so were considered unsuitable for eating behavior experiments (*47*). Consequently, for eating behavior studies (where we hypothesized that there would be a greater effect size), we compared wild-type and heterozygous Labradors, and for energy expenditure studies (where we hypothesized that there would be a smaller effect size), homozygous-affected and wild-type FCRs were used.

### Cellular models

HEK293 (XX female) cells were cultured in high-glucose Dulbecco’s modified Eagle’s medium (Gibco, catalog no. 41965039) with 1% GlutaMAX (Thermo Fisher Scientific, catalog no. 35050087), 10% fetal bovine serum (Gibco, catalog no. 10270) and penicillin (100 U/ml)/streptomycin (100 μg/ml) (Gibco, catalog no. 15140148). Cells were incubated at 37°C in humidified air containing 5% CO_2_.

### Studies in dogs

#### Dog recruitment, inclusion criteria, and obesity phenotype measurement

Owners of Labrador and FCRs were contacted by posting on social media, by email invitations sent from the U.K. Kennel Club and breed societies, and by contacting owners of suitable dogs who had participated in earlier genetic studies.

Inclusion and exclusion criteria were set in advance of dog recruitment, including requirements for age, sex, neuter status, and adiposity (described below). Dogs were excluded if they were on treatment with drugs that could affect appetite or weight (e.g., corticosteroids or anti-seizure medication), were under veterinary care for diagnosis or treatment of ill health, had donated blood within 2 months, or had recently had a significant change in weight. Dogs meeting the eligibility criteria were genotyped for the *POMC* deletion. Where more dogs were available of a given genotype than predicted required by power calculations, dogs were included on the basis of the order owners volunteered.

Weight and adiposity were measured by veterinary professionals or trained researchers using calibrated weight scales and BCS, a well-validated, ordinal measure of body fat mass scored using haptic and visual clues according to a standard set of descriptors, which are shown in fig. S2 (*24*, *25*). On this nine-point scale 1 that represents severe emaciation, four to five are considered the optimal body weight and each point above four has been shown to correspond to an ~8% increase in body fat mass when validated against measurements made using dual x-ray absorptiometry (*24*, *25*, *48*). Shoulder height was measured at the wither in standing dogs using a dog measuring stick.

#### Genotyping of the POMC deletion

DNA extracted from saliva samples was genotyped using a TaqMan assay using custom-designed primers (forward: AGGCCTTCCCCGTCGAGTTC; reverse: TACTCCAGGTCGGCCAGCG) and probes (wild-type AGGGCCCGGCCGCG with VIC fluorophore and minor groove binder (MGB) quencher; deletion TCGGCCCCGGGCGT with FAM fluorophore and MGB quencher). TaqMan gene expression master mix (Thermo Fisher Scientific, catalog no. 4369016, UK) was used with the addition of 3% dimethyl sulfoxide (Thermo Fisher Scientific, catalog no. D12345), primers at 0.4 μM, probes at 0.1 μM, and genomic DNA (0.4 ng/μl). Thermocycling was performed on an Applied Biosytems (catalog no. 4329001) 7900HT Fast Real-Time PCR System with the following thermocyle: 2 min at 50°C, 10 min at 95°C, and then 40 cycles of 15 s at 95°C followed by 1 min and 30 s at 65°C. Analysis was performed with cycle threshold (*C*_T_) set at 0.04 for wild type and 0.09 for the deletion to account for different fragment lengths. If the difference between *C*_T_ values for the two probes was >0.5, then genotype was confirmed by agarose gel separation.

#### Population level effects of POMC mutation on Adiposity in FCR

We previously reported the effect of the *POMC* mutation on weight and food motivation (*5*), the latter measured using an owner-reported measure of eating behavior, the Dog Obesity Risk Assessment questionnaire (*27*), However, we did not have BCS data for those dogs. Since that report, we have collected further FCR samples allied to BCS assigned by veterinary staff, which were analyzed for this study. All dogs included were FCR, healthy (no known or suspected systemic illness), not on treatment with medications that might alter appetite or body weight (such as anti-epilepsy drugs or corticosteroids), not being treated for orthopedic disease, at least 1 year old, and had been weighed and body condition scored for the study by a veterinary professional.

Of 221 dogs meeting the inclusion criteria, 28 were wild type, 90 were heterozygous, and 103 were homozygous for the *POMC* deletion. There were 106 male (43 neutered) and 115 female (45 neutered) with mean age of 4.6 years (range, 1 to 10), BCS of 5 (range, 4 to 7), weight of 31.0 kg (range, 15.5 to 46.0). In addition, 180 FCR had data on owner-reported food motivation with a mean of 67% (range, 12 to 100).

Linear regression was used to test for a relationship between obesity-related measures (each of BCS, weight, and food motivation score) and *POMC* genotype, age, sex, BCS, and neuter status, including interaction between sex and neuter status. The minimal model was identified using Akaike information criterion (AIC) implemented using R’s step() function. The final models included for BCS, sex × neuter status, and *POMC* deletion allele count; for body weight, sex and age and *POMC* deletion allele count; and for food motivation, neuter status and *POMC* deletion allele count.

#### Standard prefeeding

Before all experiments, dogs were introduced to a standard diet (Royal Canin Sensitivity Control Chicken and Rice canned food; nutrient composition: moisture, 72%; protein, 8.5%; fat, 6%; crude ash, 2%; crude fibers, 1%; essential fatty acids, 2.1%; metabolizable energy, 5104 kJ/kg (1198 kcal/kg); Royal Canin, Castle Cary, UK) over 7 to 10 days, eating that food exclusively for 48 hours before testing in an amount calculated to provide maintenance energy requirement (MER) calculated as 90 × (dog weight in kg)^0.75^ (kcal) divided equally into two meals, offered at 8:00 a.m. and 6:00 p.m. All dogs consumed all the food offered.

#### Measurement of incentive salience in response to a food cue (wanting)

To test wanting, we applied an inaccessible food test to measure incentive salience in response to food in 18 wild type and 18 affected Labradors (16 heterozygous and 2 homozygous for the *POMC* deletion). Of these, 10 were female, 17 were neutered, the mean age was 6.6 years (range, 0.6 to 14 years), median BCS of 5/9 (range, 4 to 7). There was no significant difference in distribution of age, sex, or BCS between genotype groups, but neutering was more common in the affected dogs (unpaired *t* test or chi-square test).

Experiments were carried out in research rooms at the Department of Veterinary Medicine. Dogs were fed half their MER 18 and 3 hours before testing, which was carried out in the morning. The owner and dog entered the room together, and the dog was allowed to explore for 1 min after which owners sat, holding the dog on a lead. A hot dog sausage was shown to the dog and then placed in a securely fastened, perforated plastic box, which was placed 2 m from the dog. The experimenter took a position 2 m from the box, opposite the owner. Owners unleashed the dog and gave a single, familiar command permitting it to manipulate the box (chosen to be familiar to the dog such as “get it!” or “it’s yours!”). Owners remained seated, still and not interacting with the dog for the 5-min duration of the experiment. Behaviors were later coded using Solomon Coder software (https://solomon.andraspeter.com/). Two independent observers used different sampling methods and different ethograms. Observer 1 was the experimenter (G.L.); observer 2 (M.D.) was blinded to the genotypes.

Observer 1 (GL) used continuous sampling with behavior occurrence expressed as a percentage of the experimental time. Coding of the behaviors was according to the following ethogram: manipulate box: dog in physical contact with the box (any body part); sit/lie down: dog in sitting or lying position (haunches touching the ground); explore: dog is away from the box and moving around the room; look at owner/experimenter/box: gazing at the owner/experimenter/box from any distance; and staying close to the owner/experimenter/box: staying within one body length to the owner/experimenter/box, or if box has moved close to people, the thing they are closest to. Data were expressed as the percentage of experimental time, during which the dog displayed each behavior.

Observer 2 (MD) applied the one-zero method of time sampling over 5-s sequential sample intervals as per the following ethogram: Sniffing: The dog has its nose close (approximately ≤5 cm) to the box without moving the box; nose push: dog moves the box by pushing it around with its nose, neither biting, licking, nor pawing it; licking: dog licks the box, tongue must be visible; pawing: one or both paws touching the box; biting: dog bites any part of the box (carrying is not classified as biting); carrying: the dog picks the box up with its mouth (either standing or walking), without throwing it in the air (playing with it); other: physical manipulation that cannot be specified because the dog is standing in a position that prohibits one from seeing what it is doing with the box (but one can still hear it is manipulating the box). Those data were reported as “interaction with box (×5-s period)”, defined as the number of 5-s periods during the 5-min recording period in which the dog was performing one or more of the above behaviors.

Comparison between genotype groups was made using unpaired *t* tests. Linear regression was additionally used to test for a relationship between food intake and *POMC* genotype, age, sex, BCS, and neuter status, and the minimal model was identified using AIC implemented using R’s step() function. Because neuter status was more common in the *POMC* deletion–affected group, regression was repeated without *POMC* genotype in the model; no effect of neutering was identified, nor was there a significant difference when an unpaired *t* test was used to compare neuter status groups.

#### Measurement of food intake at a modified ad libitum meal

To test food intake, we used a modified ad libitum meal applied to 14 wild type and 10 heterozygous *POMC* mutation Labrador retrievers. Of these, there were 8 female and 19 neutered dogs with median age of 8.5 years (range, 3 to 11), median BCS of 5/9 (range, 4 to 7/9), mean weight of 30.7 kg (range, 18.8 to 40.2). There was no significant difference between age, sex, neuter status, body weight, and BCS between genotype groups. All dogs were healthy with no clinical signs of disease or suspicion or diagnosis of systemic disease according to veterinary records.

After standardized pretest feeding, dogs were fed ½ their MER at 6:00 p.m. the night before the experiment and testing commenced at 8:00 a.m. the following morning. Testing occurred at the owners’ homes with dogs fed in their habitual location and bowls, in separate rooms/kennels to any other dogs. The experimenter observed all meals, but there was no encouragement to eat from owner or experimenter. Dogs were offered 840 g (2 cans) of the test diet at 8:30 a.m. followed by 420 g every 20 min until the trial end. The trial was ended when either (i) the dog left food uneaten for 20 min, (ii) the maximum (2940 g) set in the ethical approval was reached, or (iii) if dogs vomited or regurgitated, or (iv) in one case when the owner requested feeding be stopped at 2580 g despite the dog showing continued interest in eating. The total amount of food consumed by the dog was recorded.

ANCOVA was used to assess the difference between intake of dogs with differing *POMC* genotype, taking body weight into account. The chi-square test was used to test if vomiting/regurgitation was more common in either group. Linear regression was additionally used to test for a relationship between food intake and *POMC* genotype, age, sex, and neuter status, and the minimal model was identified using AIC implemented using R’s step() function.

#### Measurement of liking response

To examine whether dogs with the *POMC* deletion had an altered hedonic response to food, dogs were offered small amounts of dog food with and without an aversive taste (lime juice).

Thirteen wild-type dogs and 11 dogs heterozygous for the *POMC* mutation were tested. The mean age was 6 years (range, 2.5 to 13), 12 of 24 were male, 22 of 24 were neutered, and the modal BCS was 5 (range, 4 to 7). There was no significant difference between the age, sex distribution, neuter status, body weight, and BCS between genotype groups.

Dogs were provided with three different foods in a predetermined semirandom order: two commercially available palatable canned dog foods [(1) “Chicken and Rice Sensitivity Control” and (2) “low fat”, both Royal Canin] and an aversive food comprising food 1 mixed with lime juice (12 ml/50 g of food), as citric acid is a taste previously reported to be aversive to dogs (*49*, *50*). Dogs were offered 50 g of each food in a semirandom order, followed by 50 g of food 1 to test whether responses were altered by having eaten already. Foods were preprepared and offered on small transparent immobile plastic trays while dogs were restrained on leads. Half of the dogs received food 1 followed by 2, the other half food 2 and then food 1. The aversive food was third, followed by food 1 as a final control to test whether any behavior change observed with the aversive food was due to being satiated by the food they had already eaten. Dogs’ feeding behavior was video-recorded from the side and coded using the Solomon coder.

For behavioral coding, variables were defined as follows: rrial start: point when food placed on the ground and the dog approached within 15 cm; time until food finished: interval from trial start to the point where all food cleared from plate (or floor next to plate if scattered); eating: continuous while the dog is picking up food by licking, biting, or moving from one piece of food to the other without hesitation; licking: tongue is protruded to lick food from the tray; biting: dog opens its mouth and closes it over food. Bites and licks can happen simultaneously and, when either, lasts >1 frame, coded only once; stopping eating: dog stops eating—hesitating, licking its mouth, grimacing, or looking up from tray; going back: the dog starts focusing on the food again and starts eating by either licking or biting; and pause: an interval bounded by stopping eating and going back. Two-way analysis of variance (ANOVA) was used to test for the effect of both food type and *POMC* genotype on eating behavior variables.

#### Energy expenditure measurement

For energy expenditure studies, dogs were FCR homozygous for either the reference allele or *POMC* deletion, aged 2 to 7 years [to prevent any age-related effects on metabolic rate (*51*)], entire (not neutered), and of optimal body weight or ≤10% overweight (BCS of 4 to 6/9).

Dogs were habituated to experimental conditions by having a mock respirometry chamber in their homes for a week before testing. Owners were instructed to encourage dogs to relax in the kennels using reward-based methods to build a positive association. Respiration measurements took place between August and April in a research room at the Department of Veterinary Medicine. Dogs were fed half of their daily MER at least 4.5 hours before starting the respirometry measurement to ensure that they were in the post-absorptive state during testing. To control for physical activity, owners were instructed to restrict the dogs’ exercise to a short biological break in the morning and then to take them for an approximately 1 hour, 4 km on-leash walk after arrival at the test center, ending an hour before commencement of measurements.

The flow-through respirometry chamber and data analysis procedure were modified from that described in (*52*). A dog transport kennel (Petmate, Vari Traditional Dog Kennel, 122 cm by 81 cm by 89 cm; catalog no. 21700) was modified as a respiration chamber. Air inlets were fitted near the base and an air outlet on the top, to which a vacuum pump (Gardener Denver Thomas, catalog no. 2660CGHI42) was attached via a flexible hose and a flow meter (Aalborg, catalog no. GFM47). All other openings of the chamber were sealed, and Plexiglas windows allowed monitoring of the dog. The airflow was constant at a rate of 94 liter/min, and a small case fan (RS components, DC Axial Fan) ensured the mixing of air inside the chamber. The chamber was fitted with a dog bed and a water bowl and a probe measuring temperature and humidity (Hanna Instruments, catalog no. HI9565). The room temperature was kept constant at approximately 20°C. The temperature inside the chamber was, on average, 23.37°C (range, 21.0 to 26.2).

During the measurement, a gas sample was directly drawn from the chamber and ducted via flexible tubing to a drying column containing calcium sulfate (Drierite, Merck, catalog no. 7778-18-9) and onward to the analyzers (Foxbox, Sable Systems, Las Vegas, USA), which measured O_2_ and CO_2_ concentrations. Every 6 min, a baseline sample of ambient air was measured for 3 min, ducting via the same drying column to analyzers with baseline room humidity measured before and after the end of the entire experimental period. Analyzers were calibrated before each measurement using pure nitrogen and a span gas (CO_2_, 3000 parts per million; O_2_, 20.5%).

Data were analyzed with the software ExpeData (Sable Systems). On the basis of gas concentration in the chamber, the consumed volume of O_2_ (VO_2_) and the produced volume of CO_2_ (VCO_2_) were calculated (after correcting for barometric pressure, water vapor pressure, airflow rates, and gas concentrations in the ambient air). MR was calculated using the following formula as per Lighton (*53*): MR = V̇O_2_ (*l*/*h*) × [16 + 5.164 (RQ)], where RQ was calculated as the ratio of VCO_2_ to VO_2_.

An experimenter closely monitored the dog during measurement and recorded if it was resting (lying prone with head on floor) or active. Resting metabolic rate was calculated by taking the average of the lowest continuous MR values for a period of 5 min, with periods selected based on the condition that the dog was sleeping or resting and had done so for at least 5 min beforehand. The person performing data selection and analysis was blinded to the dogs’ genotype. On average, measurements lasted for 4 hours (range, 2 to 5 hours), during which dogs rested for an average duration of 1.5 hours (range, 8 min to 2.5 hours). If dogs had been restless or showed signs of distress, the measurement would have been abandoned, but this did not occur. Owners were present throughout. The study population consisted of 19 dogs and is described in Table 1.

**Table 1.** Dogs included in the energy expenditure study.

*POMC* genotype	Body mass (kg)	Age (years)	Sex	BCS (mode, range)/9	Duration rest at metabolic rate measurement (min), mean, range, first quartile, and third quartile	Respiratory quotient (mean, range)	*N*
**Wild type**	30.93, (26.2–35.05)	5.3 years, 2–7	5 F, 4 M	5 4–6	25, 5–77, 13, 30	0.86, 0.80–0.99	9
**Homozygous deletion**	31.9 (28.5–39.2	4.1, 2–6	6 F, 4 M	5, 4–6	32, 7–83, 18, 42	0.86, 0.79–0.98	10

ANCOVA was used to compare resting energy expenditure between genotype groups, adjusting for body weight. In addition, linear regression was used to model the relationship between resting energy expenditure and putative variables (*POMC* genotype, weight, age, sex, BCS, duration resting at time MR reported, and average daytime temperature for the month before measurements recorded). The minimum model was determined using the step() function in R.

#### Blood pressure measurement

Blood pressure was measured in dogs involved in eating behavior and energy expenditure experiments. Measurements were taken in dogs sitting or lying calmly in dorsal or lateral recumbency using a cuff on the antebrachium, metatarsal, or tail using the previously validated PetMAP+ II oscillometric monitor (Ramsay Medical, catalog no. 7300) (*54*). A minimum of eight repeated measurements were taken, and the highest and lowest were discarded; the mean of remaining measures is reported.

Suitable recordings were available from 21 Labradors including 13 wild type and 8 heterozygous for the *POMC* mutation, with 11 male, 17 neutered, mean age of 8.5 years (range, 3 to 12 years), and mean BCS of 5.3 (range, 4 to 7). Labrador blood pressures were measured before feeding trials in the home environment. Data from 16 FCR included 8 wild-type and 8 homozygous *POMC* deletion dogs, of which 6 were male, all were entire, mean age of 4.9 years (range 2–7), and mean BCS of 5.1 (range, 4 of 6), and all of which were measured in our clinical research facility before energy expenditure measurement. There was no significant difference between the distribution of those variables between genotype groups for either breed.

Linear regression was used to test across both breeds and within each breed for a relationship between blood pressure and *POMC* genotype, age, sex, BCS, neuter status, and breed, and the minimal model was identified using AIC implemented using R’s step() function.

#### Blood biochemistry and hematology testing

Phlebotomy was performed for clinical purposes in 11 FCR dogs from which residual blood volume was available for research purposes. Samples were obtained in the late morning (10:00 am to 12:00 pm). Each dog underwent complete blood count performed using the Sysmex XT2000i, which has been previously validated for use in dogs (*55*), and serum biochemistry testing (including sodium, potassium, chloride, bicarbonate, Na:K, anion gap, urea, creatinine, glucose, total protein, albumin, globulin, albumin:globulin ratio, calcium, phosphate, and alanine aminotransferase, alkaline phosphatase, and lipase activity) performed on an Olympus AU480 analyzer (Olympus Europa, Hamburg, Germany) using standard spectrophotometric assays for these analytes.

Plasma was separated by centrifugation immediately after sampling into EDTA as coagulant and transferred to dry ice for transport before storage at −70°C until testing. ACTH concentrations were measured using enzyme-linked immunosorbent assay (ELISA) using a commercially available kit according to the manufacturer’s instructions (Canine ACTH ELISA, Biomerica, catalog no. 7023). For thyroid hormone testing, blood was collected into plain tubes and allowed to coagulate for 2 min before separating and transporting frozen as for plasma. Canine TSH was measured using a commercial ELISA kit without modifying the manufacturer’s instructions (Canine TSH ELISA, Demeditec, catalog no. DEV9955). Total T_4_ was measured using a radioimmunoassay competition assay according to the manufacturer’s instructions (Total T_4_ RIA Kit, Beckman Coulter, catalog no. IM1447). Briefly, samples and calibrators were incubated with 125I-labeled T_4_, as tracer, in antibody-coated tubes. After incubation, the liquid content of tubes was aspirated and the bound radioactivity was determined in a gamma counter. A standard curve was constructed, and unknown values were obtained from the curve by interpolation.

### Studies in cellular models

#### Characterization of ligand-dependent responses at melanocortin receptors

For cAMP measurement assay, *MC4R* and *MC3R* constructs in pcDNA3.1(+) vector (Invitrogen, catalog no. V79020) were used through the study. Human receptor plasmids contained an N-terminal FLAG tag and canine receptor plasmids contained an N-terminal c-Myc tag. All constructs were verified with capillary sequencing. Measurement of ligand-induced cAMP generation in human embryonic kidney–293 cells was achieved using a GloSensor cAMP assay according to the manufacturer’s protocols (Promega). Briefly, 30,000 cells were plated per well of a 96-well, poly-D-lysine–coated plate. After overnight incubation, cells were transfected with both -20F cAMP reporter plasmid (100 ng per well; Promega, catalog no. E1171) and of MCR plasmid [30 ng per well; human or canine *MC3R* or *MC4R* with pcDNA3.1(+) backbone was used as a control] using Lipofectamine 2000 transfection reagent (Invitrogen, catalog no. 11668019) diluted according to the manufacturer’s instructions in Opti-MEM–reduced serum medium (Gibco, catalog no. 31985062).

The day after transfection, cell medium was replaced with 90 μl of complete media with 2% GloSensor substrate (Promega, catalog no. E1290). This was left to incubate for 2 hours at 37°C, and 5% CO_2_ before measurements were made using a Tecan SPARK Multimode Microplate reader (Tecan). Baseline measurements were recorded over 10 min with cycles at 30-s intervals before ligand activation with increasing concentrations of α-MSH (identical for human and dog; Bachem, catalog no. H-1075), human β-MSH (for human receptors; catalog no. H-1475), canine β-MSH (for canine receptors; Bachem custom synthesis) or NDP–α-MSH (as a comparator for ligand-specific responses; Bachem, catalog no. H-1100), all diluted in 0.1% bovine serum albumin and 1 mM acetic acid. After shaking, 60 luminescence readings were taken at 30-s intervals with incubation conditions maintained as above.

The total peak area under the curve values were calculated for each agonist concentration plotted as sigmoidal dose response curves with variable slope (three-parameter logistic regression) with responses to α-MSH and β-MSH normalized compared to and expressed as a percentage of the response of NDP–α-MSH. Analysis was carried out in GraphPad Prism 8 (GraphPad Software, San Diego, CA, USA). Results are from three to four independent experiments.

For β-arrestin recruitment assay, coupling between MC4R and β-arrestin 2 was measured using a NanoBiT protein:protein interaction assay (Promega, M2014). Cells were plated as for cAMP assay and transfected using Lipofectamine (as above) with MCRs cloned into a pBiT1.1-C [TK/LgBiT] vector and β-arrestin 2 cloned into a pBiT2.1-N [TK/SmBiT] vector. The cloned β-arrestin 2 gene was human, which has 97% amino acid sequence identity with canine β-arrestin 2. The positive control was achieved with SmBiT-PRKACA and LgBiT-PRKAR2A vectors and a negative control by substituting the SmBiT–β-arrestin 2 construct for a negative control vector containing HaloTag-SmBiT (Promega).

The day after transfection, cell medium was substituted for OptiMem (70 μl per well) 30 min before the assay with cells maintained at 37°C in 5% CO_2_ throughout. Luminescence was measured using a Tecan SPARK Multimode Microplate reader (Tecan). Background luminescence was established with five readings at 30-s intervals before adding Nano-Glo Live Cell Assay System reagent (25 ml per well; Promega, N2013) and establishing baseline luminescence with two measurements at 30-s intervals. Ligands were applied as for cAMP assays and shaking followed by 60 readings at 30-s intervals. Data analysis was as described for cAMP above.
