# Supplementary material for: Low resting metabolic rate and increased hunger due to β-MSH and β-endorphin deletion in a canine model
Source: Sci Adv. 2024 Mar 6;10(10):eadj3823. doi: 10.1126/sciadv.adj3823 (PMC10917344; doi:10.1126/sciadv.adj3823)
Supplement: Supplementary file 1 — Figs. S1 and S2 Table S1 Legend for movie S1 [file sciadv.adj3823_sm.pdf]

Science Advances
AAAS

Supplementary Materials for

Low resting metabolic rate and increased hunger due to β-MSH and β-endorphin deletion in a canine model

Marie T. Dittmann *et al.*

Corresponding author: Eleanor Raffan, er311@cam.ac.uk

Sci. Adv. **10**, eadj3823 (2024)
DOI: 10.1126/sciadv.adj3823

The PDF file includes:

Figs. S1 and S2
Table S1
Legend for movie S1

Other Supplementary Material for this manuscript includes the following:

Movie S1

Supplementary Material

Figure S1: α-MSH and β-MSH activate melanocortin 3 receptor similarly in both dogs and humans. *A luciferase based GloSensor cAMP assay was used to measure MC3R activation in transiently transfected HEK293 cells. The dose response following stimulation with α-MSH and β-MSH is expressed in comparison to that in response to NDP-α-MSH for both human (A) and canine (B) MC4R (both n = 3). Coupling between MC3R and β-arrestin was monitored using a NanoBiT protein:protein interaction assay in HEK293 cells transiently transfected with MC3R and luciferase activity following activation by α-MSH and β-MSH compared to that with NDP-α-MSH for both human (C) and canine (D) receptors (both n = 3 or 4). (E) EC50 values of each assay to compare potency of α-MSH and β-MSH: individual points plotted with line at median value. Statistical values displayed are unpaired student t-tests (n=3 or 4). (F) E_{max} values for each assay. Individual values with median lines plotted (n=3 or 4). Statistical values displayed are p values from unpaired student t-tests.*

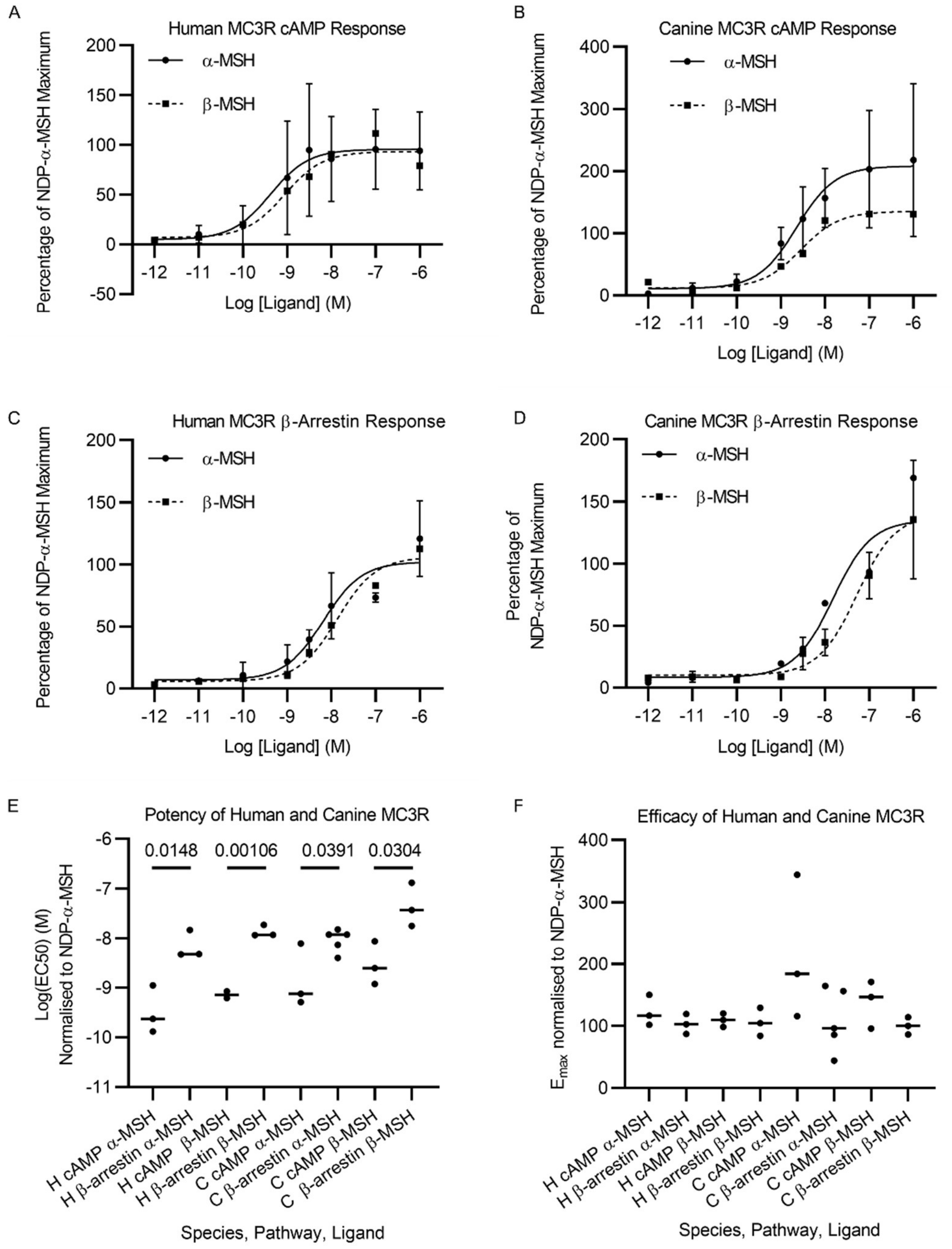

Figure S2: Body Condition Score (BCS) descriptors used to measure adiposity.

BCS were measured by veterinary professionals or trained researchers using using haptic and visual clues according to a standard set of descriptors which are shown below. On this 9 point scale 1 represents severe emaciation, 4-5 are considered optimal body weight and each point above 4 has been shown to correspond to an ~8% increase in body fat mass (24, 25, 48).

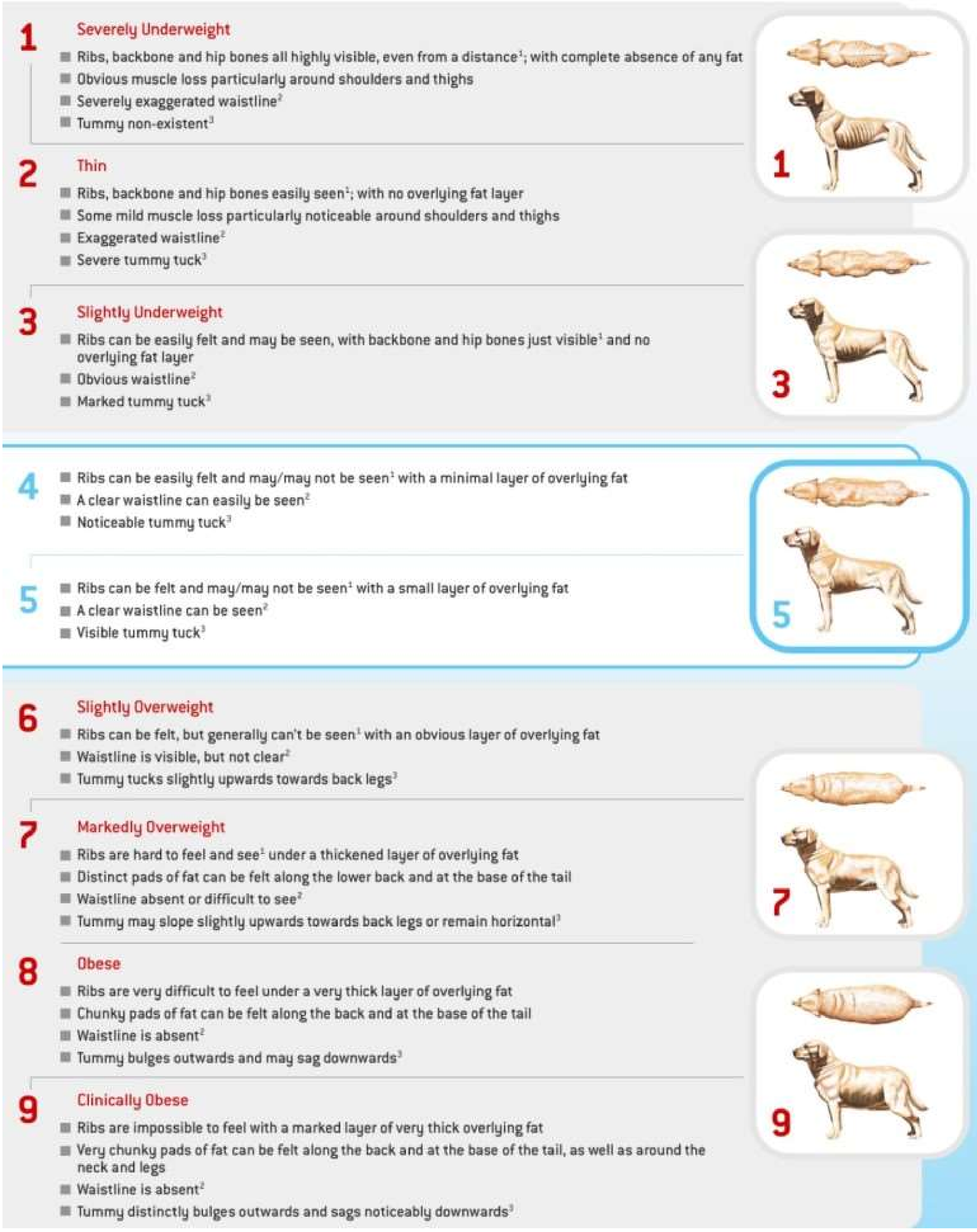

Table S1: Endocrine testing in dogs of contrasting genotype for the POMC Deletion

ACTH (pg/mL)							
POMC Genotype	*No. of Dogs*	*Mean*	*Standard Deviation*	*Median*	*Interquartile Range*	*Minimum*	*Maximum*
Wild Type	*4*	*17.3*	*7.84*	*16.4*	*9.67*	*9.3*	*27*
Heterozygous	*4*	*19.5*	*2.12*	*19.5*	*1.5*	*18*	*21*
Homozygous deletion	*5*	*21.5*	*2.52*	*21*	*3*	*18.6*	*25*

tT4 (nmol/L)	*Clinical reference range: 13 – 52 nmol/L*						
POMC Genotype	*No. of Dogs*	*Mean*	*Standard Deviation*	*Median*	*Interquartile Range*	*Minimum*	*Maximum*
Wild Type	*4*	*15.4*	*2.01*	*14.8*	*2.23*	*13.8*	*18.2*
Heterozygous	*4*	*15.0*	*2.98*	*14.6*	*1.8*	*11.9*	*19.1*
Homozygous deletion	*5*	*21.6*	*5.64*	*25*	*10.5*	*15.3*	*26*

TSH (ng/mL)	*Clinical reference range: <0.42 ng/mL*						
POMC Genotype	*No. of Dogs*	*Mean*	*Standard Deviation*	*Median*	*Interquartile Range*	*Minimum*	*Maximum*
Wild Type	*4*	*0.185*	*0.0681*	*0.17*	*0.055*	*0.12*	*0.28*
Heterozygous	*4*	*0.27*	*0.133*	*0.28*	*0.125*	*0.1*	*0.42*
Homozygous deletion	*5*	*0.26*	*0.207*	*0.19*	*0.11*	*0.12*	*0.62*

Supplementary Video 1: Representative clips of video recordings of dogs of contrasting genotype performing the inaccessible food task to test motivational salience in response to a food cue. *Brief methodological summary is also included.*
